# Genomic characterization of *Listeria monocytogenes* isolated from small ruminants in integrated crop-livestock systems

**DOI:** 10.1186/s12866-025-04626-9

**Published:** 2026-01-06

**Authors:** Sejin Cheong, Joanna G. Rothwell, Cory Schlesener, Bart C. Weimer, Craig Miramontes, Richard V. Pereira, Paulo Pagliari, Alda F. A. Pires

**Affiliations:** 1 Department of Population Health and Reproduction, School of Veterinary Medicine, University of California -Davis, Davis, CA USA; 2 Department of Soil, Water, and Climate, College of Food, Agriculture and Natural Resources Sciences, University of Minnesota, Saint Paul, MN USA

**Keywords:** Whole-genome sequencing, Virulence factor, Antimicrobial susceptibility testing, *Listeria monocytogenes*, Integrated crop-livestock systems

## Abstract

*Listeria monocytogenes* (*L. monocytogenes*), a foodborne pathogen shed by asymptomatic ruminants, poses a contamination risks in integrated crop-livestock systems (ICLS), where ruminants are introduced to graze cover crops prior to replanting fresh produce in a field. As a follow-up study, we conducted whole genome sequencing of 30 *L. monocytogenes* isolates obtained from sheep and goat fecal and soil samples collected during our previously published ICLS field trial studies (2019—2022) at organic farms in California and Minnesota. One goat fecal isolate was genetically identical to one soil isolate collected at seven days post-grazing. Most isolates (28/30, 93.3%) belonged to lineage I, specifically to serogroup IVb or IVb-v1, and were classified as CC1 or CC554, both clonal complexes previously associated with human listeriosis outbreaks. The majority of isolates harbored virulence associated genes, including LIPI-1 or LIPI-3 genes, and *agrA*, associated with biofilm formation and survival in agricultural soils. In antimicrobial susceptibility testing, all isolates were susceptible to antibiotics commonly used to treat ruminant and human listeriosis, including ampicillin and penicillin. These findings suggest that asymptomatic small ruminants in ICLS may introduce pathogenic *L. monocytogenes* strains into the agricultural environment, posing a potential contamination risk to fresh produce despite the low overall prevalence observed.

**Supplementary Information:**

The online version contains supplementary material available at 10.1186/s12866-025-04626-9.

## Introduction

*Listeria monocytogenes* (*L. monocytogenes*) is a foodborne pathogenic bacterium that can survive and persist in diverse environments, including feces of animals, soil, and vegetation [1–3]. Listeriosis in humans remains a significant public health and clinical concern due to its fatal outcomes such as septicemia, central nervous system infections, and abortion [4]. Most reported human cases have been linked to the consumption of contaminated dairy or meat products [4–6]. However, asymptomatic shedding of *L. monocytogenes* by healthy animals, including cattle, sheep, and goats, has been documented, contributing to environmental contamination on farms [7, 8]. Although infections linked to fresh produce contaminated via animal feces and soil in vegetable farms are relatively rare, *L. monocytogenes* presence in growing environments represents a significant food safety concern [6, 9]. Indirect contamination of *L. monocytogenes* from livestock to fresh produce has been associated with human outbreaks; for instance, sheep manure was identified as the source of *L. monocytogenes* outbreak in cabbage used for coleslaw [10].

With the increasing application of whole genome sequencing (WGS) to trace transmission routes of *L. monocytogenes* for surveillance and outbreak investigation, understanding potential sources of contamination in complex farm environments has become more feasible [5, 11–13]. Previously, serotyping (e.g., 1/2a, 1/2b, 4b) or serogrouping (e.g., IVb, IVb-v1, IIa, IIb) based on specific antisera has been performed over decades despite its relatively low discriminatory power [14]. Recently, however, phylogenetic analyses including multilocus sequence typing (MLST) using seven housekeeping gene fragments and WGS have enabled identification of four distinct lineages (I–IV), and further subdivided into clonal complexes (CCs) and sequence types (STs) [15]. Each ST represents a unique allelic profile of the seven housekeeping genes, and CCs group STs that differ by a single locus [16], thereby enabling more detailed strain comparisons. For example, Dreyer et al. (2015) investigated the transmission of *L. monocytogenes* between sheep and the farm environment by performing three genetic subtyping methods and confirmed that identical strains were isolated from sheep, soil, and water tanks in a farm [17]. Furthermore, genomic analyses including virulence genes, antimicrobial resistance genes, and mobile genetic elements enable the identification of the genetic diversity and evolution of *L. monocytogenes*, which may explain its wide distribution in the environment and among different hosts [13, 18]. However, the extent of fecal shedding of *L. monocytogenes* from small ruminants and its persistence in agricultural farm environments has not been extensively investigated [9, 15, 19]. In particular, limited studies have focused on asymptomatic small ruminants [7, 8, 20], although some studies have examined small ruminant cases with clinical symptoms, such as neurological disorders [21–23].

As one of the regenerative farming practices, integrated crop-livestock systems (ICLS) involve grazing small ruminants on cover crops grown between fresh produce planting cycles [24, 25]. While raw manure can serve as an organic fertilizer, it poses a potential risk of transferring *L. monocytogenes* from asymptomatic grazing animals to fresh vegetable growing environments [26, 27]. In our previous four-year ICLS field trials (2019—2022), sheep and goats were directly introduced into fields to graze cover crops prior to planting tomatoes, spinach, and cucumbers [28, 29]. *L. monocytogenes* was detected in 0.5% (1/220) of sheep fecal samples from California (CA), and in 21.9% (35/160) of goat fecal samples and 0.2% (1/540) of soil samples collected in Minnesota (MN). *L. monocytogenes* was rarely found in soil and was not detected in any vegetable samples in our previous studies. The single positive soil sample detected in MN may have originated from the small ruminants. However, no high-resolution methods were applied at that time to confirm this.

To build upon the findings of our previously published studies [28, 29], the present study conducted a follow-up investigation to further characterize 30 *L. monocytogenes* isolates obtained from the previous ICLS field trials. Specifically, this study aimed to 1) assess the genetic similarity of *L. monocytogenes* isolated from fecal and soil samples detected, and 2) characterize *L. monocytogenes* isolates from the feces of asymptomatic sheep and goats used for grazing, using WGS data. Given the limited research on asymptomatic small ruminants, this study contributes to filling a knowledge gap regarding *L. monocytogenes* isolates from healthy small ruminants in agricultural settings.

## Materials and methods

### Sample collection and L. monocytogenes isolation methods

A total of 30 *L. monocytogenes* isolates including 28 from goat feces, one from sheep feces, and one from soil, were obtained during crop-livestock integration field trials (2019—2022) in CA and MN, as described in our two previous studies [28, 29]. Of the 28 isolates from goat feces, 24 isolates were obtained in 2022, 16 from pre-graze and eight from post-graze treatment groups (Table 1). The two previous studies were field trials with a randomized complete block design with four replicates. Three treatments (fallow as a control, cover crop tilled without grazing, cover crop grazed by sheep or goats) were randomly assigned to each block. Following the cropping sequence generally adopted by each region, cover crop mix was seeded (November in CA, August/September in MN), and grazing was started when the cover crop height was approximately 25 cm in the grazed treatment plots. Depending on the size of plots (4.5 × 121.9—236.2 m in [28] and 4.5 × 22.9 m in [29] per one paddock with four replicates) and amount of biomass in each year, 25—120 sheep in CA and 115—170 goats in MN grazed the cover crops. Fecal samples were collected both before grazing from the barn or trailer floors and after grazing from the plots immediately after sheep or goat were removed from the grazed plots. Briefly, to isolate *L. monocytogenes*, fecal (10 g) and soil (30 g) samples were enriched in Listeria Enrichment Broth (90mL and 270mL, respectively) (Neogen Culture Media, Lansing, MI), followed by Immunomagnetic separation (IMS) [30]. A 30 μL aliquot of the IMS washed bead product was plated on Brillance Listeria Agar (BLA) with selective supplements (Oxoid, Hants, United Kingdom), while 100 μL of the IMS product was also added into Fraser broth (BD, Sparks, MD). Both media were incubated for 48 h at 37 °C. After the sequential isolation process, presumptive positive colonies (blue colonies with a halo) were streaked onto Tryptic Soy Agar (TSA). *L. monocytogenes* were confirmed via conventional PCR targeting the *hylA* gene [31]. Isolates from each positive sample were preserved in 15% glycerol with Tryptic Soy Broth at −80 °C. For further regrowth for DNA extraction and antimicrobial susceptibility testing, one isolate was selected from each sample to ensure that each isolate corresponded to an individual sample.

**Table 1:** Isolates of *L. monocytogenes* (*n* = 30) from crop-livestock integration field trials (2019—2022). Sheep were used as grazing animals in California (CA), while goats were used in Minnesota (MN)

Isolate ID	Sample Type	Treatment group	State	Year	Serogroup (SG)	Sequence Type (ST)	Clonal Complex (CC)	Lineage
BCW14370	Feces	Post-graze	CA	2019	IIa,IIb, or IIc	918	CC918	II
BCW14357	Feces	Pre-graze	MN	2022	IIb or L	1965	CC1283	III
BCW14381	Feces	Post-graze	MN	2022	IVb	4	CC4	I
BCW14359	Feces	Pre-graze	MN	2022	IVb	4	CC4	I
BCW14376	Feces	Pre-graze	MN	2022	IVb	4	CC4	I
BCW14364	Feces	Pre-graze	MN	2022	IVb	4	CC4	I
BCW14379	Feces	Post-graze	MN	2022	IVb	4	CC4	I
BCW14356	Feces	Pre-graze	MN	2022	IVb	4	CC4	I
BCW14380	Feces	Post-graze	MN	2022	IVb	4	CC4	I
BCW14368	Feces	Pre-graze	MN	2021	IVb	219	CC4	I
BCW14365	Feces	Pre-graze	MN	2020	IVb	1	CC1	I
BCW14369	Feces	Pre-graze	MN	2021	IVb-v1	554	CC554	I
BCW14366	Feces	Pre-graze	MN	2020	IVb-v1	554	CC554	I
BCW14385	Feces	Post-graze	MN	2022	IVb-v1	554	CC554	I
BCW14384	Feces	Post-graze	MN	2022	IVb-v1	554	CC554	I
BCW14373	Feces	Pre-graze	MN	2022	IVb-v1	554	CC554	I
BCW14372	Feces	Pre-graze	MN	2022	IVb-v1	554	CC554	I
BCW14355	Feces	Pre-graze	MN	2022	IVb-v1	554	CC554	I
BCW14375	Feces	Pre-graze	MN	2022	IVb-v1	554	CC554	I
BCW14360	Feces	Pre-graze	MN	2022	IVb-v1	554	CC554	I
BCW14374	Feces	Pre-graze	MN	2022	IVb-v1	554	CC554	I
BCW14358	Feces	Pre-graze	MN	2022	IVb-v1	554	CC554	I
BCW14386	Soil	D7 Post-graze	MN	2022	IVb-v1	554	CC554	I
BCW14371	Feces	Pre-graze	MN	2022	IVb-v1	554	CC554	I
BCW14354	Feces	Pre-graze	MN	2022	IVb-v1	554	CC554	I
BCW14383	Feces	Post-graze	MN	2022	IVb-v1	554	CC554	I
BCW14377	Feces	Post-graze	MN	2022	IVb-v1	554	CC554	I
BCW14378	Feces	Post-graze	MN	2022	IVb-v1	554	CC554	I
BCW14361	Feces	Pre-graze	MN	2022	IVb-v1	554	CC554	I
BCW14363	Feces	Pre-graze	MN	2022	IVb-v1	554	CC554	I

## Whole-genome sequencing

Isolates stored in 15% glycerol cryotubes were plated on TSA for regrowth and incubated overnight at 37 °C. A 10 μL loop of colonies was inoculated into Mueller–Hinton Broth (MHB) (MilliporeSigma, Carlsbad, CA) and incubated overnight. A total of 1.5 mL of MHB culture was transferred into two microcentrifuge tubes and centrifuged at 13,000—16,000 × g for 5 min. Genomic DNA was extracted from the resulting pellets using the Wizard genomic DNA purification kit (Promega Corporation, Madison, WI, USA), following the manufacturer’s instructions for gram positive bacteria, with slight modifications. Briefly, 20 μL of lysozyme and mutanolysin were substituted at the lytic enzyme addition step and following the first incubation step 15 μL of proteinase K was added and incubated at 65 °C for 10 min. The resulting DNA pellet at the end of protocol was rehydrated with Tris HCl solution overnight at 4 °C. DNA concentration (ng/μL) and purity (A260/280 ≥ 1.8 and A260/A230 ≥ 2.0 used as thresholds for the next step) were quantified using a Nanodrop One UV–Vis Spectrophotometer (ThermoScientific, Waltham, MA, USA). Genomic DNA integrity was assessed using TapeStation (Agilent 4200, Santa Clara, CA, USA) before storage at −20 °C until WGS [32, 33].

WGS was conducted following the established 100 K Pathogen Genome Project protocol [34–36]. Sequencing libraries were prepared from high-quality genome DNA through enzymatic fragmentation and size selection (average 450 bp inserts), then sequenced using the Illumina HiSeq X platform (San Diego, CA, USA). Raw sequence data with all accession numbers on the NCBI database are available in the Supplementary Material.

## Genome assembly and analyses

Whole genome sequence data were processed with Trimmomatic (version 0.39) to remove low-quality sequence and sequencing adapters [37], using settings “PE ILLUMINACLIP: < adapters > :2:40:15 LEADING:2 TRAILING:2 SLIDINGWINDOW:4:15 MINLEN:50” with program provided Illumina adaptor sequences (“https://github.com/timflutre/trimmomatic/tree/master/adapters”, accessed August 1 st, 2022). Quality of sequence data was assessed by FastQC (version 0.12.1) [38], and samples were removed from analysis if they failed any of the following modules 'Adapter Content', 'Per base sequence quality', 'Per sequence quality scores', and 'Sequence Duplication Levels'.

Contamination by PhiX internal sequencing standard was searched for by Kraken2 (version 2.1.3) [39] and if found, removed by read alignment with Bowtie2 (version 2.5.3) [40], both using the Illumina PhiX reference sequence (https://webdata.illumina.com/downloads/productfiles/igenomes/phix/PhiX_Illumina_RTA.tar.gz, accessed August18, 2020). Microbial taxonomy and contamination were assessed with Kraken2 and Bracken (version 2.9) [41]. Kraken2 microbial database was constructed using the microbial genomes from NCBI RefSeq database (Kraken2 program preset reference libraries archaea, bacteria, viral, fungi, protozoa, and UniVec_Core, downloaded/constructed August 2nd, 2023); Bracken database was built off Kraken2 microbial database by standard protocol, using the parameters of k-mer size = 35 and read size = 150. Contamination seen by Kraken2/Bracken was defined as > 5% of reads matching another species; samples identified as *Listeria monocytogenes* were used for analysis.

Genome assemblies were constructed with Shovill (version 1.1.0) [42] using the default options with the SPAdes assembler [43]. Genome assembly quality was reviewed with CheckM (version 1.2.2), using the “lineage_wf” workflow [44]. Each assembly’s depth of coverage was measured with Mosdepth (version 0.3.6) [45], using the “fast-mode” setting, and Bowtie2 and Samtools (version 1.19.2) for trimmed reads alignment to assembly preparation [46]. Quality control cutoffs for inclusion in the analyses were CheckM: > 90% estimated completeness, < 6% estimated contamination, within range 2.5–3.5 Mbases for assembly size, < 300 contigs; Mosdepth: > 30 × mean depth of coverage.

Whole genome similarity comparisons were performed using Sourmash (version 4.8.6) with a K-mer size of 31 [47], and a Sourmash heatmap plot was created with a scale of 100,000 k-mers per megabase (scaled sketch size of 10) to assess the genomic relatedness of each *L. monocytogenes* isolates.

### *In silico* genomic characterization of *L. monocytogenes* isolates - multilocus sequence typing, virulence associated genes, stress survival islets, biofilm formation related genes, antimicrobial resistance (AMR) genes, and plasmid profiles

Serogroups (SGs) were manually assigned according to the Doumith’s scheme based on the presence/absence pattern of specific marker genes—*lmo0737*, *lmo1118*, *ORF2110*, *ORF2819*, and *prs* identified in each assembled genome [48]. Multilocus sequence typing (MLST) was performed using seven housekeeping genes (*abcZ*, *bglA*, *cat*, *dapE*, *dat*, *ldh*, and *lhkA*) to assign sequence types (STs) with the “mlst” program (version 2.23.0) [49] using “listeria_2” schema of the PubMLST database [50] (database packaged with program, updated 2022 Oct 28). Clonal complexes (CCs) and lineages were inferred from MLST profiles using the *L. monocytogenes* database hosted by the Institut Pasteur. Genes of interest further listed were searched for in genome assemblies by ABRicate (version 1.0.1) [51], with a standard set of databases (downloaded 2023 Nov 4). Virulence-associated genes were identified by screening against the Virulence Factor Database (VFDB) [52]. The presence of stress survival islets and the *sigB* (*lmo0895*) gene was assessed to examine stress tolerance potential, while biofilm formation genes (agrA, agrC, and luxS), potentially associated with soil attachment and adaptation, were identified using the *L. monocytogenes* database from the Institut Pasteur. AMR genes were detected using multiple databases: ARG-ANNOT [53], the Comprehensive Antibiotic Resistance Database (CARD) [54], MEGARes [55], the NCBI AMR database [56], and ResFinder [57]. The presence of plasmids was assessed using PlasmidFinder [58].

## Phenotypic antimicrobial susceptibility testing

Isolates preserved in 15% glycerol cryotubes were plated onto the TSA for resuscitation and BLA to assess potential contamination, followed by overnight incubation at 37°C. *L. monocytogenes* colonies from TSA plates were submitted to the California Animal Health and Food Safety (CAHFS) Laboratory for antimicrobial susceptibility testing. Testing was conducted using the broth microdilution method with the Sensititre Vet Bovine/porcine plate (BOPO7F) (Thermo Fisher Scientific, Wilmington, DE). As the official breakpoints used to define the susceptibility and resistance profiles of *L. monocytogenes* are limited to penicillin, ampicillin and trimethoprim–sulfamethoxazole in the Clinical and Laboratory Standards Institute (ICLS M100 ED33) and the European Committee on Antimicrobial Susceptibility Testing (EUCAST), interpretations for clindamycin and gentamicin were made using breakpoints established for *Staphylococcus spp.*, recommended by previous studies [59, 60]. For the other antimicrobials without any breakpoint established, minimum inhibitory concentrations (MIC) were reported.

## Statistical analysis

Descriptive statistics were used to summarize the distribution of MLST types, virulence factors, stress survival islets, biofilm formation, AMR genes, and plasmid presence across all isolates. Since pre- and post-graze fecal samples were not collected from the same individual animals, Fisher’s exact test was performed to evaluate the differences in the presence of virulence-associated genes between the treatment groups (pre- and post-graze), with significance set at *p* < 0.05. A heatmap was generated in R (v.4.3.0) with the ggplot2 package to visualize the distribution of virulence-associated genes.

## Results

### MLST typing and whole genome comparison of isolates

The draft genome consists of 22—60 contigs with a coverage depth of 95 ×—845 × and is estimated > 99% complete based on the quality control. WGS of *L. monocytogenes* (30 isolates) identified six STs and CCs by MLST (Table 1). Most isolates (28/30, 93.3%) belonged to lineage I, specifically to serogroup IVb or IVb-v1, and were assigned to CC1 (ST1), CC4 (ST4 or ST219), and CC554 (ST554). The hypervirulent CC1 and CC4 were detected in goat fecal samples collected both pre- (6 isolates) and post-graze (3 isolates) treatments across 2020—2022. In contrast, one isolate identified from a sheep fecal sample belonged to lineage II and was assigned to CC918 (ST918), showing a distinct genetic profile compared to goat fecal isolates.

**Fig. 1:**
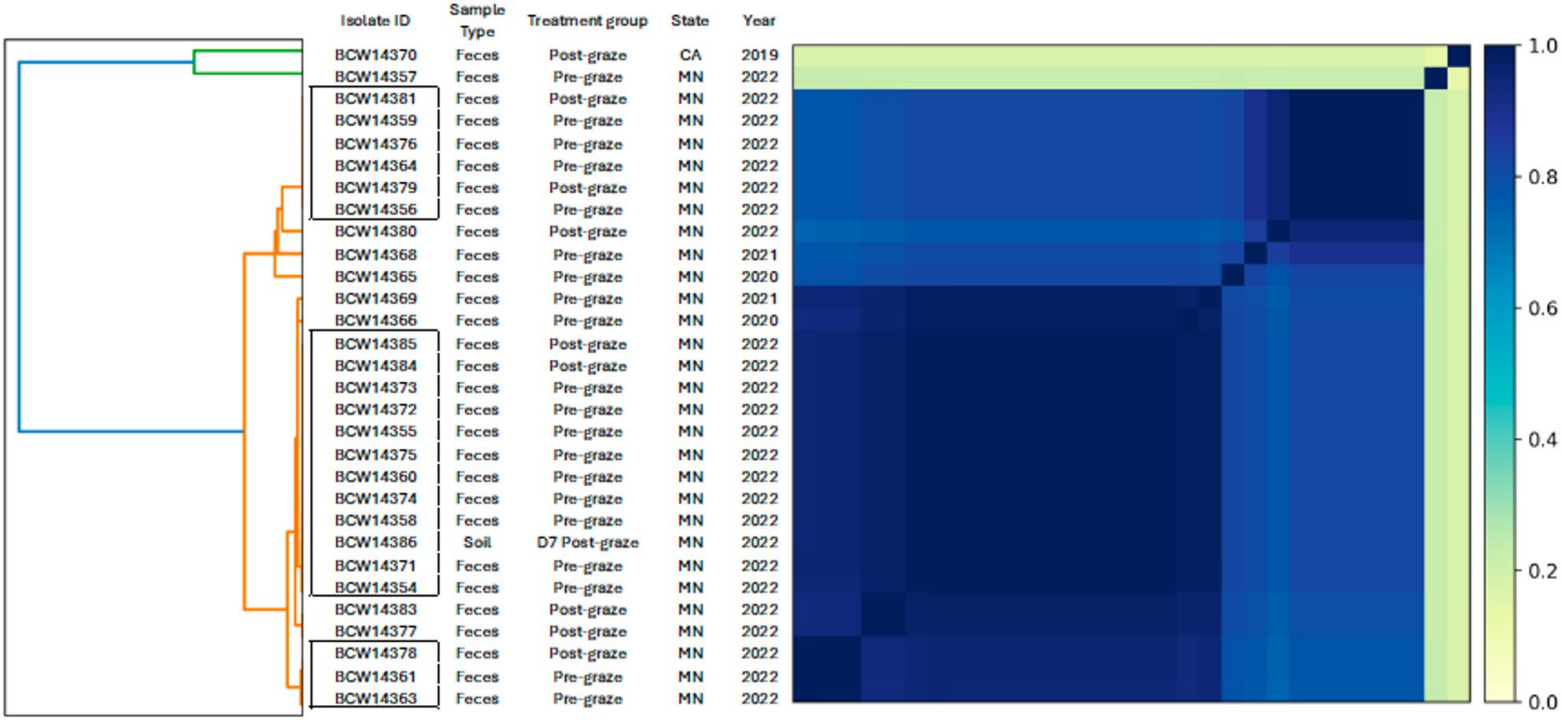
Whole-genome similarity heatmap of *L. monocytogenes* isolates (*n* = 30) collected from crop-livestock integration field trials (2019—2022). The heatmap visualizes pairwise genomic similarities based on the Jaccard similarity index with the color gradient on the right indicating the relative degree of similarity (0–1)

WGS comparisons identified three clusters of isolates, each displaying complete pairwise genomic similarity (Jaccard index = 1) (Fig. 1). Two of these clusters were identified as CC554 (ST554), the most prevalent CCs/STs. One soil-derived *L. monocytogenes* isolate (isolate ID: BCW14386), collected at 7 days after goat grazing of the field, showed identical genomic similarity to pre- and post-graze goat fecal isolates collected in the same year. Although this soil isolate was obtained from a non-grazed treatment plot, adjacent to the grazed plot in our previous field trial, its genetic similarity suggests potential transfer from the grazed plot. Interestingly, two pre-graze fecal isolates collected in 2020 (Isolate ID: BCW14366) and 2021 (Isolate ID: BCW14369) showed high genomic similarity to the isolates identified in 2022, with all classified as CC554 (ST554).

### Profiles of virulence-associated, stress tolerance, and biofilm-related genes in L. monocytogenes isolates

**Fig. 2:**
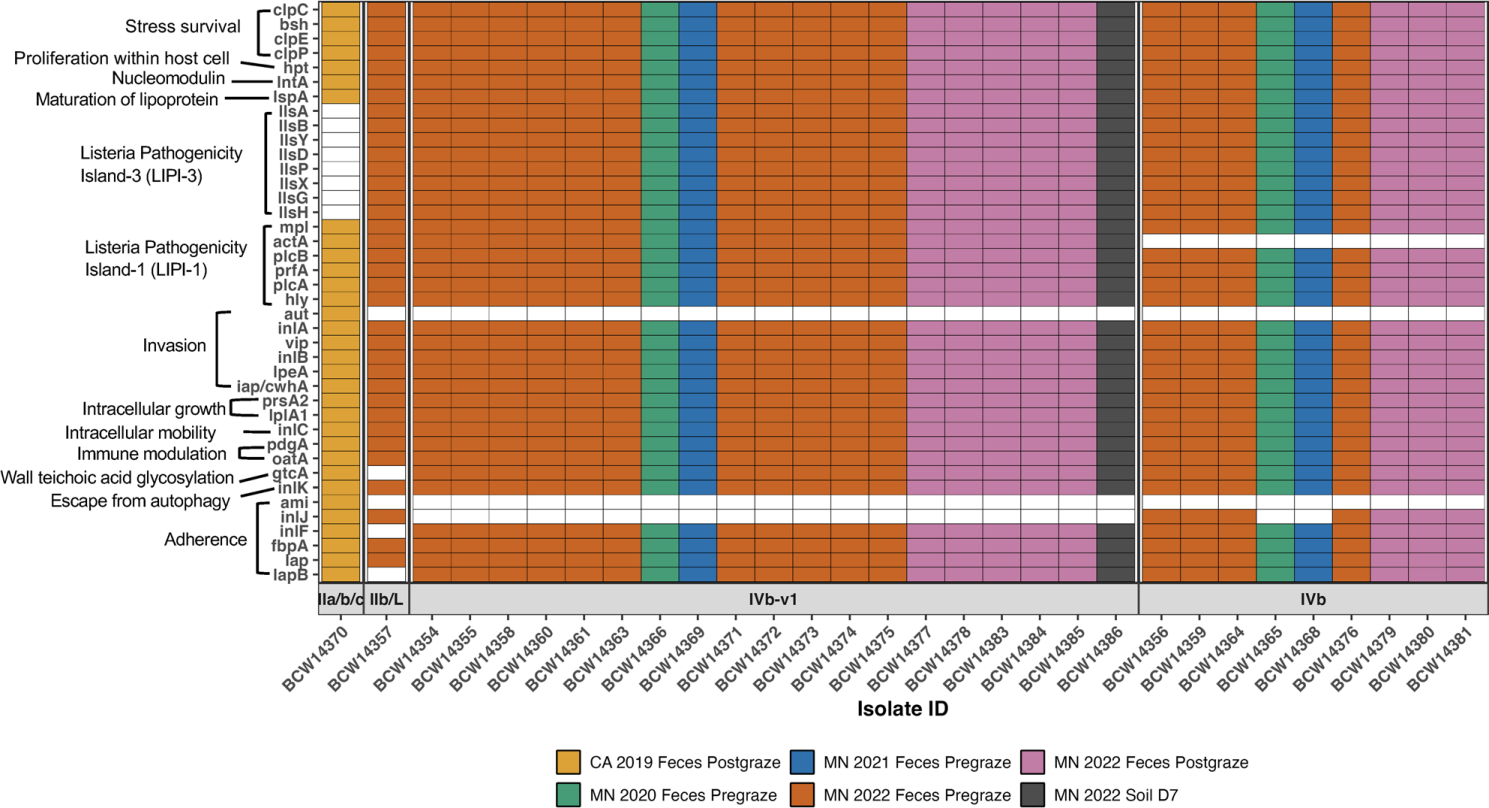
Distribution of virulence-associated genes in *L. monocytogenes* isolates categorized by state, year, and treatment (pre-/post- graze) group (indicated by color) and serogroup (grouped in box), based on Virulence Factor Database analysis. Absence of color indicates that the gene was not detected

The profiles of virulence-associated genes in *L. monocytogenes* isolates were generally consistent within each serogroup (Fig. 2). Most isolates, except for the nine isolates classified as serogroup IVb, contained the entire *Listeria* Pathogenicity Island-1 (LIPI-1), which includes the *prfA, plcA, plcB, actA, mpl, and hly* genes. A key difference between isolates belonging to serogroups IVb and IVb-v1 was the presence or absence of the *inlJ* or *actA* genes. The *actA* gene, a component of LIPI-1, encodes the actin assembly-inducing protein, which facilitates intercellular mobility and cell-to-cell spread during infection [61]. The *InlJ* gene encodes a fibronectin-binding protein associated with bacterial adherence [62]. Additionally, all 29 isolates identified from goats and soil contained *Listeria* Pathogenicity Island-3 (LIPI-3), which encodes listeriolysin S and comprises eight genes: *llsA, llsG, llsH, llsX, llsB, llsY, llsD, llsP*. However, one isolate from sheep did not have all LIPI-3 genes.

Analysis of isolates by pre- and post-graze treatment groups revealed that most isolates in each group belonged to serogroup IVb-v1 and carried all the LIPI-1 and LIPI-3 genes. Specifically, 65% (13/20) in the pre-graze and 55.6% (5/9) in the post-graze treatment groups contained these virulence factors. No statistical difference was observed between pre- and post-graze fecal samples regarding the presence of the *inlJ* or *actA* genes.

All isolates contained stress survival genes listed in the VFDB, including *clpC, clpE,* and *clpP* (encoding stress proteins)*,* and *bsh* (related to bile salt hydrolase production) (Fig. 2). Interestingly, one isolate from sheep feces carried stress survival islet 1 (SSI-1), comprising five genes – *lmo0444, lmo0445, lmo0446 (pva), lmo0447 (gadD1), and lmo0448 (gadT1)*. In contrast, none of the isolates from goats or soils contained these SSI-1 genes, which are known to confer tolerance to environmental stress such as acid, salt, and high osmolality. Additionally, the master regulator *sigB* gene, involved in stress survival gene regulations, was present in all isolates, except one (BCW14357). Regarding biofilm-associated genes, *agrA, agrC, and luxS* were detected in all the isolates except BCW14357, which lacked *agrC* but carried *agrA* and *luxS*.

### AMR genes and AMR phenotypes

All 30 *L. monocytogenes* isolates shared identical antibiotic resistance gene profiles, including *fosX* (fosfomycin)*, mprF* (defensin)*, Lin* (lincomycin)*, lmo0919_fam* (lincomycin), *norB* (fluoroquinolone), and *MDrL.* The *MDrL* is associated with multidrug efflux mechanisms in *L. monocytogenes*, facilitating the export of macrolide-based antibiotics and cefotaxime [63]. The phenotypic antimicrobial susceptibility testing results are summarized in Table 2. According to CLSI (≤ 2µg/mL; susceptible) or EUCAST (≤ 1µg/mL; susceptible) breakpoints, all isolates were susceptible to ampicillin or penicillin. Due to the presence of a single MIC range on the testing plate, the susceptibility of TMS (Trimethoprim/Sulfamethoxazole) could not be determined. When assessed the other antibiotics using CLSI breakpoints for *Staphylococcus spp.*, all isolates demonstrated intermediate susceptibility (by CLSI; ≥ 4µg/mL; resistant) or resistant (by EUCAST; ≥ 0.25µg/mL; resistant) to clindamycin and were susceptible to gentamycin (by CLSI; ≤ 4µg/mL; susceptible, by EUCAST; ≤ 2µg/mL; susceptible).

**Table 2:** Phenotypic antimicrobial susceptibility testing results with minimum inhibitory concentrations (MIC) of 30 *L. monocytogenes* isolates. Antibiotics in blue represent those with established breakpoints by CLSI or EUCAST, but the other remaining antibiotics are either mostly used in veterinary medicine or lack defined breakpoints

	Number of isolates with MIC (µg/mL) (out of 30 isolates in total)												
Antibiotics	≤ 0.125	0.25	0.5	1	2	4	8	16	32	64	128	≥ 256	MIC range
Ampicillin		9	18	3									0.25–16
Ceftiofur^*^							4	26					0.25–8
Chlortetracycline^*^			1	26	3								0.5–8
Clindamycin^+^				1	29								0.25–16
Danofloxacin^*^					30								0.12–1
Enrofloxacin^*^				3	27								0.12–2
Florfenicol^*^					2	28							0.25–8
Gentamicin +				30									1–16
Neomycin						30							4–32
Oxytetracycline^*^				6	24								0.5–8
Penicillin	6	4	18	2									0.12–8
Sulphadimethoxine^*^												33	256
Spectinomycin								1	28	1			8–64
Tiamulin^*^									1	29			1–32
Tilmicosin^*^								10	20				4–64
TMS (Trimethoprim/Sulfamehtoxazole)					30								2/38
Tulathromycin^*^							30						8–64
Tylosin^*^						29	1						0.5–32

^*^Antibiotics are used in veterinary medicine for livestock in the US

^+^Breakpoints established for *Staphylococcus spp.* were used for defining the susceptibility and resistance

### Detection of plasmids

Plasmid sequences were found in 20% (6/30) of the *L. monocytogenes* isolates. Three different types of plasmids — LMIVRS16815, pS86, pEF1071 — were identified across six *L. monocytogenes* genomes isolated from pre- and post- graze fecal samples collected in 2022. Among these, five isolates harbored LMIVRS16815, three carried pS86, and one isolate (BCW14363) contained all three plasmids.

## Discussion

Over the four-year (2019—2022) ICLS field trial conducted in CA and MN, we observed a higher number of *L. monocytogenes* positive cases in goat fecal samples in MN compared to those from sheep in CA [28, 29]. None of the grazing animals showed clinical symptoms such as neurological signs. One soil isolate collected from MN was genetically identical to goat fecal isolates collected in the same year based on whole genome comparisons. This finding suggests potential contamination of *L. monocytogenes* from feces to soil, with possible spread to an adjacent block via wind or other environmental vectors, as the isolate was detected in a non-grazed adjacent block. However, given that the proportion of *L. monocytogenes*-positive soil samples collected from MN was 0.2% (1/540), and that this soil isolate was detected only at 7 days post grazing and was not afterwards over 143 days post grazing, our results indicate that *L. monocytogenes* did not persist in this agricultural environment after contamination events occurred. [29].

We further characterized the genetic diversity, lineage classification, and potential genetic risk profiles of the isolates based on clonal relationships, virulence genes, and antimicrobial resistance genes. Except for the two isolates, one from sheep (lineage II) and one from goats (lineage III), all other 28 isolates in this study belonged to lineage I. This finding contrasts with the established understanding of *L. monocytogenes* evolutionary lineages: isolates from natural environmental sources or food products (e.g., ready-to-eat foods, dairy products) have been associated with lineage II, while lineage I strains are more commonly linked to severe clinical listeriosis cases in both humans and ruminants [16, 18, 64]. Similarly, studies conducted in Europe by Dreyer et al. (2016) and Papić et al. (2019) reported that *L. monocytogenes* isolates from natural environmental sources, such as feed, manure, soil and healthy ruminant feces, were significantly associated with lineage II, whereas isolates from ruminant clinical cases of rhombencephalitis or neurolisteriosis were predominantly associated with lineage I [20, 23]. In contrast, studies from the US reported that both lineage I and II isolates were commonly identified from neurologic and bacteremia cases in ruminants [22], and that lineage II was the most frequently associated with ruminant listeriosis with clinical manifestation [21]. Nevertheless, our results indicate that lineage I is not restricted to clinical cases but can also be present in healthy small ruminants and their surrounding environmental samples.

Regarding the clonal complexes (CCs) of the goat fecal isolates in this study, isolates were identified as CC1 (one isolate), CC4 (nine isolates), or CC554 (19 isolates). Similarly, Papić et al. (2019) found that CC1 and CC4 were the most prevalent CCs among isolates from both animal clinical cases and natural environmental sources [20]. Palacios-Gorba (2021) also reported that CC1 and CC4 were the most frequent CCs in fecal samples from ruminants, including cattle, sheep, and goats [19]. In a study analyzing the distribution of CCs among serotype 4b *L. monocytogenes* isolates from North America, CC4 was significantly overrepresented in human clinical isolates compared to food and other sources [65]. Furthermore, in a study integrating human epidemiological and clinical data with bacterial population genomics, both CC1 and CC4 were recognized as hypervirulent CCs and strongly associated with human clinical infections [66]. Interestingly, CC554 (ST554) was identified in a listeriosis outbreak in Illinois and Michigan in 2014, linked to fresh produce (mung bean sprouts), which had not been previously reported in listeriosis outbreak at that time [10]. Therefore, considering that the majority of isolates identified in this study belong to CC554 and CC4, there is a potential for these *L. monocytogenes* strains to pose a public health risk if transferred to fresh produce [29]. However, none of the produce samples tested positive, and only one soil sample (7 days after grazing) was positive in this study, suggesting that limited *L. monocytogenes* transfer occurred from ruminant feces to the ICLS environment.

*Listeria* Pathogenicity Islands (LIPIs) are key gene clusters in *L. monocytogenes* that contribute to its virulence, as they encode essential virulence factors commonly found in pathogenic strains [12]. All isolates in this study contained the complete LIPI-1 (*prfA, plcA, plcB, actA, mpl, and hly* genes), which is involved in the intracellular infection cycle, although a few isolates lacked the *actA* gene. LIPI-3, encoding a listeriolysin S, was absent from a sheep fecal sample (lineage II), but was detected in all goat fecal isolates, which were all classified as lineage I. This observation is consistent with previous reports that LIPI-3 is exclusively associated with lineage I [67]. The widespread presence of LIPI-1 and restricted distribution of LIPI-3 among lineage I strains have also been observed in earlier genomic studies of *L. monocytogenes* from ruminants and ready-to-eat foods in the US [21, 68]. These findings confirm that the isolates derived from these asymptomatic grazing ruminants possessed the key gene cluster profiles generally associated with *L. monocytogenes* pathogenicity.

Regarding virulence-associated genes, a key distinguishing feature between isolates belonging to serogroups IVb (classified as CC1 or 4) and IVb-v1 (classified as CC554) in goat fecal samples was the differential presence of the *inlJ* or *actA* genes. The *inlJ* gene, a recently identified member of the internalin family related to anchoring to the cell wall, is conserved in pathogenic serovars of *L. monocytogenes* and serves as a virulence determinant [62, 69, 70]. Accordingly, seven isolates belonging to serogroups IVb, known to be more hypervirulent, harbored the *inlJ* gene in this study, while none of the isolates in the serogroup IVb-v1 contained it. In contrast, the 19 isolates classified as serogroup IVb-v1 harbored the *ActA* gene, which enables actin polymerization and cell-to-cell spread, thereby contributing to bacterial aggregation and biofilm formation [61]. Notably, serogroup IVb-v1 comprised the majority of goat fecal isolates in this study, and was the only serogroup consistently detected across all three years of sampling (2020–2022).

Environmental survival and persistence of *L. monocytogenes* are facilitated by its ability to form biofilms and the presence of stress tolerance genes such as SSI-1 [71–73]. In this study, SSI-1 was detected in only one isolate obtained from a sheep fecal sample, whereas none of the isolates from goats carried this islet gene. Keeney et al. (2018) reported that the presence of SSI-1 is serotype-dependent and associated with strong biofilm formation [74]. Specifically, serotype 1/2b (serogroup IIb) predominantly harbored SSI-1 and formed strong biofilms, while serotype 4b (serogroups IVb or IVb-v1) typically lacked SSI-1 and showed weak biofilm formation. This serotype-specific distribution may explain the absence of SSI-1 among the goat isolates in this study, which were mostly classified as serogroups IVb or IVb-v1. Instead, we found that all isolates, except one, contained the *agrA* genes. The *agr* communication system regulates adhesion and biofilm formation and is essential for optimal survival of *L. monocytogenes* in soil [75]. However, most isolates (29/30) also harbored *luxS,* which is known to be associated with the inhibition of attachment and biofilm development [76].

The antibiotic resistance genes identified from this study (i.e., *fosX*, *mprF, Lin, lmo0919, norB*, and *MDr*L) and phenotypic antimicrobial susceptibility testing results showed consistent patterns across all 30 isolates. All isolates demonstrated resistance against fosfomycin (fosX), lincomycin (Lin), and fluoroquinolone (norB), which aligns with findings from other studies investigating *L. monocytogenes* isolates from ruminants, fresh produce, or wildlife samples in the US [21, 77, 78]. The presence of the *fosX* gene across all isolates was expected, as *L. monocytogenes* is known to be naturally resistant to Fosfomycin [79]. All isolates harbored the *Lin* gene and showed intermediate susceptibility or resistance to clindamycin in phenotypic testing, both of which are associated with the lincosamide class. Notably, none of the isolates exhibited resistance to penicillin or ampicillin, in agreement with previous studies [78, 80, 81]. Given that penicillin or ampicillin alone or in combination with gentamicin is recommended as the first-line therapy for human listeriosis [82], the observed susceptibility of all isolates to these antibiotics is clinically favorable. This is also significant from a One Health standpoint, demonstrating that these livestock-derived *L. monocytogenes* isolates retained susceptibility to antimicrobial agents used to treat both human and ruminant listeriosis cases (e.g., penicillin, ampicillin and oxytetracycline).

This study has some limitations. Fecal samples were collected from the field immediately after grazing events, across different blocks of the field, but within the same flock. Therefore, it is possible that some *L. monocytogenes* isolates originated from the same individual animal, which may have contributed to the high genomic similarity among them. Additionally, only one *L. monocytogenes* isolate was recovered from sheep fecal samples over the three-year period. This limited our ability to conduct meaningful genomic comparisons between sheep and goat isolates. Phenotypic antimicrobial susceptibility testing was performed using veterinary antimicrobial panels, which did not include several antibiotics widely used in human medicine (e.g., ciprofloxacin, tetracycline). Furthermore, standardized MIC breakpoints for veterinary antibiotics in *L. monocytogenes* are not well-established, complicating the interpretation of resistance results. Lastly, although unlikely, some genes may appear to be missing in our genome analysis due to annotation differences between our data and the reference databases.

## Conclusions

By conducting WGS of *L. monocytogenes* isolates obtained from the ICLS field trials, genomic characterization indicated that goat fecal isolates may pose a potential public health risk if transferred to fresh produce grown in ICLS fields. Most of the isolates belonged to CC1 and CC554, both of which have been relevant to human listeriosis outbreaks. The isolates also harbored virulence associated genes, including LIPI-1 or LIPI-3 genes related to intracellular infection and survival within hosts, and *agrA* genes associated with biofilm formation and enhanced persistence in the agricultural soil. However, all isolates were susceptible to penicillin, ampicillin, and gentamicin, suggesting that they remain treatable with commonly used human and ruminant antimicrobial agents, an important consideration when reviewing the results from a One Health perspective. Since one soil isolate was genetically identical to goat fecal isolates collected in the same year based on whole genome comparisons, which implies that fecal isolates from asymptomatic goats could be potentially transferred to soil and survive up to seven days post grazing. Although we could not find enough isolates from sheep feces due to the practical constraints of grazing implementation in real world ICLS settings, further genomic investigation of *L. monocytogenes* isolates from asymptomatic sheep is needed.

## Supplementary Information

Supplementary Material 1.

## Data Availability

Raw sequence data with the accession numbers on the NCBI database are provided within the supplementary information.

## References

[1] Dhama K, Karthik K, Tiwari R, Shabbir MZ, Barbuddhe S, Malik SVS, et al. Listeriosis in animals, its public health significance (food-borne zoonosis) and advances in diagnosis and control: a comprehensive review. Vet Q. 2015;35:211–35. 10.1080/01652176.2015.1063023. 26073265 10.1080/01652176.2015.1063023

[2] Oevermann A, Zurbriggen A, Vandevelde M. Rhombencephalitis caused by *Listeria monocytogenes* in humans and ruminants: a zoonosis on the rise? Interdiscip Perspect Infect Dis. 2010;2010:1–22. 10.1155/2010/632513. 10.1155/2010/632513 PMC2829626 20204066

[3] Freitag NE, Port GC, Miner MD. *Listeria monocytogenes* — from saprophyte to intracellular pathogen. Nat Rev Microbiol. 2009;7:623–8. 10.1038/nrmicro2171. 19648949 10.1038/nrmicro2171 PMC2813567

[4] Koopmans MM, Brouwer MC, Vázquez-Boland JA, Van De Beek D. Human listeriosis. Clin Microbiol Rev. 2023;36:e00060–19. 10.1128/cmr.00060-19. 36475874 10.1128/cmr.00060-19 PMC10035648

[5] Cabal A, Pietzka A, Huhulescu S, Allerberger F, Ruppitsch W, Schmid D. Isolate-based surveillance of *Listeria monocytogenes* by whole genome sequencing in Austria. Front Microbiol. 2019;10:2282. 10.3389/fmicb.2019.02282. 31632381 10.3389/fmicb.2019.02282 PMC6779813

[6] Cartwright EJ, Jackson KA, Johnson SD, Graves LM, Silk BJ, Mahon BE. Listeriosis outbreaks and associated food vehicles, United States, 1998–2008. Emerg Infect Dis. 2012;:1–9. 10.3201/eid1901. 10.3201/eid1901.120393 PMC3557980 23260661

[7] Nightingale KK, Schukken YH, Nightingale CR, Fortes ED, Ho AJ, Her Z, et al. Ecology and transmission of *Listeria monocytogenes* infecting ruminants and in the farm environment. Appl Environ Microbiol. 2004;70:4458–67. 10.1128/AEM.70.8.4458-4467.2004. 15294773 10.1128/AEM.70.8.4458-4467.2004 PMC492327

[8] Esteban JI, Oporto B, Aduriz G, Juste RA, Hurtado A. Faecal shedding and strain diversity of *Listeria monocytogenes* in healthy ruminants and swine in Northern Spain. BMC Vet Res. 2009;5:2. 10.1186/1746-6148-5-2. 19133125 10.1186/1746-6148-5-2 PMC2651128

[9] Walland J, Lauper J, Frey J, Imhof R, Stephan R, Seuberlich T, et al. *Listeria monocytogenes* infection in ruminants: is there a link to the environment, food and human health? A review. SAT. 2015;157:319–28. 10.17236/sat00022. 26753347 10.17236/sat00022

[10] Garner D, Kathariou S. Fresh produce-associated listeriosis outbreaks, sources of concern, teachable moments, and insights. J Food Prot. 2016;79:337–44. 10.4315/0362-028X.JFP-15-387. 26818997 10.4315/0362-028X.JFP-15-387

[11] Van Walle I, Björkman JT, Cormican M, Dallman T, Mossong J, Moura A, et al. Retrospective validation of whole genome sequencing-enhanced surveillance of listeriosis in Europe, 2010 to 2015. Eurosurveillance. 2018;23. 10.2807/1560-7917.ES.2018.23.33.1700798. 10.2807/1560-7917.ES.2018.23.33.1700798 PMC6205253 30131096

[12] Wiktorczyk-Kapischke N, Skowron K, Wałecka-Zacharska E. Genomic and pathogenicity islands of *Listeria monocytogenes*—overview of selected aspects. Front Mol Biosci. 2023;10:1161486. 10.3389/fmolb.2023.1161486. 37388250 10.3389/fmolb.2023.1161486 PMC10300472

[13] Moura A, Criscuolo A, Pouseele H, Maury MM, Leclercq A, Tarr C, et al. Whole genome-based population biology and epidemiological surveillance of *Listeria monocytogenes*. Nat Microbiol. 2016;2:16185. 10.1038/nmicrobiol.2016.185. 27723724 10.1038/nmicrobiol.2016.185 PMC8903085

[14] Seeliger HPR, Höhne K. Chapter II Serotyping of Listeria monocytogenes and Related Species. In: Bergan T, Norris JR, editors. Academic Press; 1979. p. 31–49. 10.1016/S0580-9517(08)70372-6.

[15] Bagatella S, Tavares-Gomes L, Oevermann A. *Listeria monocytogenes* at the interface between ruminants and humans: a comparative pathology and pathogenesis review. Vet Pathol. 2022;59:186–210. 10.1177/03009858211052659. 34856818 10.1177/03009858211052659

[16] Haase JK, Didelot X, Lecuit M, Korkeala H, Achtman M. The ubiquitous nature of Listeria monocytogenes clones: a large-scale Multilocus Sequence Typing study. Environ Microbiol. 2014;16:405–16. 10.1111/1462-2920.12342. 24274459 10.1111/1462-2920.12342

[17] Dreyer M, Thomann A, Böttcher S, Frey J, Oevermann A. Outbreak investigation identifies a single *Listeria monocytogenes* strain in sheep with different clinical manifestations, soil and water. Vet Microbiol. 2015;179:69–75. 10.1016/j.vetmic.2015.01.025. 25726302 10.1016/j.vetmic.2015.01.025

[18] Orsi RH, Bakker HCD, Wiedmann M. *Listeria monocytogenes* lineages: genomics, evolution, ecology, and phenotypic characteristics. Int J Med Microbiol. 2011;301:79–96. 10.1016/j.ijmm.2010.05.002. 20708964 10.1016/j.ijmm.2010.05.002

[19] Palacios-Gorba C, Moura A, Gomis J, Leclercq A, Gómez-Martín Á, Bracq-Dieye H, et al. Ruminant-associated *Listeria monocytogenes* isolates belong preferentially to dairy-associated hypervirulent clones: a longitudinal study in 19 farms. Environ Microbiol. 2021;23:7617–31. 10.1111/1462-2920.15860. 34863016 10.1111/1462-2920.15860

[20] Papić B, Pate M, Félix B, Kušar D. Genetic diversity of *Listeria monocytogenes* strains in ruminant abortion and rhombencephalitis cases in comparison with the natural environment. BMC Microbiol. 2019;19:299. 10.1186/s12866-019-1676-3. 31849320 10.1186/s12866-019-1676-3 PMC6918561

[21] Cardenas-Alvarez MX, Zeng H, Webb BT, Mani R, Muñoz M, Bergholz TM. Comparative genomics of *Listeria monocytogenes* isolates from ruminant listeriosis cases in the Midwest United States. Microbiol Spectr. 2022;10:e01579–22. 10.1128/spectrum.01579-22. 36314928 10.1128/spectrum.01579-22 PMC9769944

[22] Steckler AJ, Cardenas-Alvarez MX, Townsend Ramsett MK, Dyer N, Bergholz TM. Genetic characterization of *Listeria monocytogenes* from ruminant listeriosis from different geographical regions in the U.S. Vet Microbiol. 2018;215:93–7. 10.1016/j.vetmic.2017.12.021. 29290393 10.1016/j.vetmic.2017.12.021

[23] Dreyer M, Aguilar-Bultet L, Rupp S, Guldimann C, Stephan R, Schock A, et al. *Listeria monocytogenes* sequence type 1 is predominant in ruminant rhombencephalitis. Sci Rep. 2016;6:36419. 10.1038/srep36419. 27848981 10.1038/srep36419 PMC5111077

[24] Hilimire K. Integrated crop livestock agriculture in the United States: a review. J Sustain Agric. 2011;35:376–93. 10.1080/10440046.2011.562042.

[25] Lemaire G, Franzluebbers A, Carvalho PCdeF, Dedieu B. Integrated crop–livestock systems: strategies to achieve synergy between agricultural production and environmental quality. Agric Ecosyst Environ. 2014;190:4–8. 10.1016/j.agee.2013.08.009.

[26] Ho AJ, Ivanek R, Gröhn YT, Nightingale KK, Wiedmann M. *Listeria monocytogenes* fecal shedding in dairy cattle shows high levels of day-to-day variation and includes outbreaks and sporadic cases of shedding of specific *L. monocytogenes* subtypes. Prev Vet Med. 2007;80:287–305. 10.1016/j.prevetmed.2007.03.005. 17481754 10.1016/j.prevetmed.2007.03.005

[27] Haley BJ, Sonnier J, Schukken YH, Karns JS, Van Kessel JAS. Diversity of *Listeria monocytogenes* within a U.S. dairy herd, 2004–2010. Foodborne Pathog Dis. 2015;12:844–50. 10.1089/fpd.2014.1886. 26325149 10.1089/fpd.2014.1886

[28] Cheong S, Jay-Russell MT, Chandler-Khayd C, Di Francesco J, Haghani V, Aminanadi P, et al. Presence of foodborne pathogens and survival of generic *Escherichia coli* in an organic integrated crop-livestock system. Front Sustain Food Syst. 2024;8:1343101. 10.3389/fsufs.2024.1343101.

[29] Cheong S, Chandler-Khayd C, Williams SR, Gaudin ACM, Aminabadi P, Jay-Russell MT, et al. Evaluation of environmental risk factors associated with survival of generic *E. coli* in organic integrated crop-livestock systems in California and Minnesota. Front Sustain Food Syst. 2024;8:1464018. 10.3389/fsufs.2024.1464018.

[30] Cooley MB, Quinones B, Oryang D, Mandrell RE, Gorski L. Prevalence of shiga toxin producing *Escherichia coli, Salmonella enterica*, and *Listeria monocytogenes* at public access watershed sites in a California Central Coast agricultural region. Front Cell Infect Microbiol. 2014;4. 10.3389/fcimb.2014.00030. 10.3389/fcimb.2014.00030 PMC3940966 24624367

[31] Kawasaki S, Horikoshi N, Okada Y, Takeshita K, Sameshima T, Kawamoto S. Multiplex PCR for simultaneous detection of *Salmonella* spp., *Listeria monocytogenes*, and *Escherichia coli* O157:H7 in meat samples. J Food Prot. 2005;68:551–6. 10.4315/0362-028X-68.3.551. 15771181 10.4315/0362-028x-68.3.551

[32] Jeannotte R, Lee E, Kong N, Ng W, Weimer BC, Kelly L. High-Throughput Analysis of Foodborne Bacterial Genomic DNA Using Agilent 2200 TapeStation and Genomic DNA ScreenTape System. Agilent Technologies Application Note. Agilent Technologies; 2014.

[33] Kong N, Ng W, Cai L, Leonardo A, Weimer BC, Kelly L. Integrating the DNA Integrity Number (DIN) to Assess Genomic DNA (gDNA) Quality Control Using the Agilent 2200 TapeStation System. Agilent Technologies Application Note. Agilent Technologies; 2016.

[34] Weimer BC. 100K pathogen genome project. Genome Announc. 2017;5:e00594-e617. 10.1128/genomeA.00594-17. 28705971 10.1128/genomeA.00594-17 PMC5511910

[35] Weis AM, Storey DB, Taff CC, Townsend AK, Huang BC, Kong NT, et al. Genomic comparison of Campylobacter spp. and their potential for zoonotic transmission between Birds, Primates, and Livestock. Appl Environ Microbiol. 2016;82:7165–75. 10.1128/AEM.01746-16. 27736787 10.1128/AEM.01746-16 PMC5118927

[36] Chen P, Den Bakker HC, Korlach J, Kong N, Storey DB, Paxinos EE, et al. Comparative genomics reveals the diversity of restriction-modification systems and DNA methylation sites in *Listeria monocytogenes*. Appl Environ Microbiol. 2017;83:e02091–16. 10.1128/AEM.02091-16. 27836852 10.1128/AEM.02091-16 PMC5244299

[37] Bolger AM, Lohse M, Usadel B. Trimmomatic: a flexible trimmer for Illumina sequence data. Bioinformatics. 2014;30:2114–20. 10.1093/bioinformatics/btu170. 24695404 10.1093/bioinformatics/btu170 PMC4103590

[38] Andrews S. A quality control tool for high throughput sequence data. 2023. https://www.bioinformatics.babraham.ac.uk/projects/fastqc/.

[39] Wood DE, Lu J, Langmead B. Improved metagenomic analysis with Kraken 2. Genome Biol. 2019;20:257. 10.1186/s13059-019-1891-0. 31779668 10.1186/s13059-019-1891-0 PMC6883579

[40] Langmead B, Salzberg SL. Fast gapped-read alignment with Bowtie 2. Nat Methods. 2012;9:357–9. 10.1038/nmeth.1923. 22388286 10.1038/nmeth.1923 PMC3322381

[41] Lu J, Breitwieser FP, Thielen P, Salzberg SL. Bracken: estimating species abundance in metagenomics data. PeerJ Comput Sci. 2017;3:e104. 10.7717/peerj-cs.104. 40271438 10.7717/peerj-cs.104 PMC12016282

[42] Seemann T. Shovill: Assemble bacterial isolate genomes from Illumina paired-end reads. 2017. https://github.com/tseemann/shovill.

[43] Bankevich A, Nurk S, Antipov D, Gurevich AA, Dvorkin M, Kulikov AS, et al. SPAdes: a new genome assembly algorithm and its applications to single-cell sequencing. J Comput Biol. 2012;19:455–77. 10.1089/cmb.2012.0021. 22506599 10.1089/cmb.2012.0021 PMC3342519

[44] Parks DH, Imelfort M, Skennerton CT, Hugenholtz P, Tyson GW. CheckM: assessing the quality of microbial genomes recovered from isolates, single cells, and metagenomes. Genome Res. 2015;25:1043–55. 10.1101/gr.186072.114. 25977477 10.1101/gr.186072.114 PMC4484387

[45] Pedersen BS, Quinlan AR. Mosdepth: quick coverage calculation for genomes and exomes. Bioinformatics. 2018;34:867–8. 10.1093/bioinformatics/btx699. 29096012 10.1093/bioinformatics/btx699 PMC6030888

[46] Danecek P, Bonfield JK, Liddle J, Marshall J, Ohan V, Pollard MO, et al. Twelve years of SAMtools and BCFtools. Gigascience. 2021;10:giab008. 10.1093/gigascience/giab008. 33590861 10.1093/gigascience/giab008 PMC7931819

[47] Titus Brown C, Irber L. Sourmash: a library for MinHash sketching of DNA. JOSS. 2016;1:27. 10.21105/joss.00027.

[48] Doumith M, Buchrieser C, Glaser P, Jacquet C, Martin P. Differentiation of the major *Listeria monocytogenes* serovars by multiplex PCR. J Clin Microbiol. 2004;42:3819–22. 10.1128/JCM.42.8.3819-3822.2004. 15297538 10.1128/JCM.42.8.3819-3822.2004 PMC497638

[49] Seemann T. mlst: Scan contig files against traditional PubMLST typing schemes. 2015. https://github.com/tseemann/mlst.

[50] Jolley KA, Bray JE, Maiden MCJ. Open-access bacterial population genomics: BIGSdb software, the PubMLST.org website and their applications. Wellcome Open Res. 2018;3:124. 10.12688/wellcomeopenres.14826.1. 30345391 10.12688/wellcomeopenres.14826.1 PMC6192448

[51] Seemann T. ABRicate: Mass screening of contigs for antimicrobial resistance or virulence genes. 2015. https://github.com/tseemann/abricate.

[52] Liu B, Zheng D, Zhou S, Chen L, Yang J. VFDB 2022: a general classification scheme for bacterial virulence factors. Nucleic Acids Res. 2022;50:D912-7. 10.1093/nar/gkab1107. 34850947 10.1093/nar/gkab1107 PMC8728188

[53] Gupta SK, Padmanabhan BR, Diene SM, Lopez-Rojas R, Kempf M, Landraud L, et al. ARG-ANNOT, a new bioinformatic tool to discover antibiotic resistance genes in bacterial genomes. Antimicrob Agents Chemother. 2014;58:212–20. 10.1128/AAC.01310-13. 24145532 10.1128/AAC.01310-13 PMC3910750

[54] Alcock BP, Huynh W, Chalil R, Smith KW, Raphenya AR, Wlodarski MA, et al. CARD 2023: expanded curation, support for machine learning, and resistome prediction at the Comprehensive Antibiotic Resistance Database. Nucleic Acids Res. 2023;51:D690–9. 10.1093/nar/gkac920. 36263822 10.1093/nar/gkac920 PMC9825576

[55] Bonin N, Doster E, Worley H, Pinnell LJ, Bravo JE, Ferm P, et al. MEGARes and AMR++, v3.0: an updated comprehensive database of antimicrobial resistance determinants and an improved software pipeline for classification using high-throughput sequencing. Nucleic Acids Research. 2023;51:D744–52. 10.1093/nar/gkac1047. 10.1093/nar/gkac1047 PMC9825433 36382407

[56] Feldgarden M, Brover V, Gonzalez-Escalona N, Frye JG, Haendiges J, Haft DH, et al. AMRFinderPlus and the reference gene catalog facilitate examination of the genomic links among antimicrobial resistance, stress response, and virulence. Sci Rep. 2021;11:12728. 10.1038/s41598-021-91456-0. 34135355 10.1038/s41598-021-91456-0 PMC8208984

[57] Bortolaia V, Kaas RS, Ruppe E, Roberts MC, Schwarz S, Cattoir V, et al. ResFinder 4.0 for predictions of phenotypes from genotypes. J Antimicrob Chemother. 2020;75:3491–500. 10.1093/jac/dkaa345. 32780112 10.1093/jac/dkaa345 PMC7662176

[58] Carattoli A, Zankari E, García-Fernández A, Voldby Larsen M, Lund O, Villa L, et al. *In silico* detection and typing of plasmids using PlasmidFinder and plasmid multilocus sequence typing. Antimicrob Agents Chemother. 2014;58:3895–903. 10.1128/AAC.02412-14. 24777092 10.1128/AAC.02412-14 PMC4068535

[59] Conter M, Paludi D, Zanardi E, Ghidini S, Vergara A, Ianieri A. Characterization of antimicrobial resistance of foodborne *Listeria monocytogenes*. Int J Food Microbiol. 2009;128:497–500. 10.1016/j.ijfoodmicro.2008.10.018. 19012982 10.1016/j.ijfoodmicro.2008.10.018

[60] Li Q, Sherwood JS, Logue CM. Antimicrobial resistance of *Listeria* spp. recovered from processed bison. Lett Appl Microbiol. 2007;44:86–91. 10.1111/j.1472-765X.2006.02027.x. 17209820 10.1111/j.1472-765X.2006.02027.x

[61] Travier L, Guadagnini S, Gouin E, Dufour A, Chenal-Francisque V, Cossart P, et al. ActA promotes *Listeria monocytogenes* aggregation, intestinal colonization and carriage. PLoS Pathog. 2013;9:e1003131. 10.1371/journal.ppat.1003131. 23382675 10.1371/journal.ppat.1003131 PMC3561219

[62] Sabet C, Lecuit M, Cabanes D, Cossart P, Bierne H. LPXTG protein InlJ, a newly identified internalin involved in *Listeria monocytogenes* virulence. Infect Immun. 2005;73:6912–22. 10.1128/IAI.73.10.6912-6922.2005. 16177371 10.1128/IAI.73.10.6912-6922.2005 PMC1230919

[63] Mata MT, Baquero F, Perez-Diaz JC. A multidrug efflux transporter in *Listeria monocytogenes*. FEMS Microbiol Lett. 2000;187:185–8. 10.1111/j.1574-6968.2000.tb09158.x. 10856655 10.1111/j.1574-6968.2000.tb09158.x

[64] Balandyte L, Brodard I, Frey J, Oevermann A, Abril C. Ruminant rhombencephalitis-associated *Listeria monocytogenes* alleles linked to a multilocus variable-number tandem-repeat analysis complex. Appl Environ Microbiol. 2011;77:8325–35. 10.1128/AEM.06507-11. 21984240 10.1128/AEM.06507-11 PMC3233052

[65] Lee S, Chen Y, Gorski L, Ward TJ, Osborne J, Kathariou S. *Listeria monocytogenes* source distribution analysis indicates regional heterogeneity and ecological niche preference among serotype 4b clones. MBio. 2018;9:e00396–18. 10.1128/mBio.00396-18. 29666282 10.1128/mBio.00396-18 PMC5904418

[66] Maury MM, Tsai Y-H, Charlier C, Touchon M, Chenal-Francisque V, Leclercq A, et al. Uncovering *Listeria monocytogenes* hypervirulence by harnessing its biodiversity. Nat Genet. 2016;48:308–13. 10.1038/ng.3501. 26829754 10.1038/ng.3501 PMC4768348

[67] Cotter PD, Draper LA, Lawton EM, Daly KM, Groeger DS, Casey PG, et al. Listeriolysin S, a novel peptide haemolysin associated with a subset of lineage I *Listeria monocytogenes*. PLoS Pathog. 2008;4:e1000144. 10.1371/journal.ppat.1000144. 18787690 10.1371/journal.ppat.1000144 PMC2522273

[68] Chen Y, Chen Y, Pouillot R, Dennis S, Xian Z, Luchansky JB, et al. Genetic diversity and profiles of genes associated with virulence and stress resistance among isolates from the 2010–2013 interagency *Listeria monocytogenes* market basket survey. PLoS ONE. 2020;15:e0231393. 10.1371/journal.pone.0231393. 32352974 10.1371/journal.pone.0231393 PMC7192433

[69] Hamon M, Bierne H, Cossart P. *Listeria monocytogenes*: a multifaceted model. Nat Rev Microbiol. 2006;4:423–34. 10.1038/nrmicro1413. 16710323 10.1038/nrmicro1413

[70] Bierne H, Cossart P. *Listeria monocytogenes* surface proteins: from genome predictions to function. Microbiol Mol Biol Rev. 2007;71:377–97. 10.1128/MMBR.00039-06. 17554049 10.1128/MMBR.00039-06 PMC1899877

[71] Hilliard A, Leong D, O’Callaghan A, Culligan E, Morgan C, DeLappe N, et al. Genomic characterization of *Listeria monocytogenes* isolates associated with clinical listeriosis and the food production environment in Ireland. Genes. 2018;9:171. 10.3390/genes9030171. 29558450 10.3390/genes9030171 PMC5867892

[72] Lakicevic BZ, Den Besten HMW, De Biase D. Landscape of stress response and virulence genes among *Listeria monocytogenes* strains. Front Microbiol. 2022;12:738470. 10.3389/fmicb.2021.738470. 35126322 10.3389/fmicb.2021.738470 PMC8811131

[73] Ryan S, Begley M, Hill C, Gahan CGM. A five-gene stress survival islet (SSI-1) that contributes to the growth of *Listeria monocytogenes* in suboptimal conditions: stress survival islet in *L. monocytogenes*. J Appl Microbiol. 2010;109:984–95. 10.1111/j.1365-2672.2010.04726.x. 20408910 10.1111/j.1365-2672.2010.04726.x

[74] Keeney K, Trmcic A, Zhu Z, Delaquis P, Wang S. Stress survival islet 1 contributes to serotype-specific differences in biofilm formation in *Listeria monocytogenes*. Lett Appl Microbiol. 2018;67:530–6. 10.1111/lam.13072. 30218533 10.1111/lam.13072

[75] Vivant A-L, Garmyn D, Gal L, Piveteau P. The Agr communication system provides a benefit to the populations of Listeria monocytogenes in soil. Front Cell Infect Microbiol. 2014;4. 10.3389/fcimb.2014.00160. 10.3389/fcimb.2014.00160 PMC4222237 25414837

[76] Sela S, Frank S, Belausov E, Pinto R. A mutation in the *luxS* gene influences *Listeria monocytogenes* biofilm formation. Appl Environ Microbiol. 2006;72:5653–8. 10.1128/AEM.00048-06. 16885324 10.1128/AEM.00048-06 PMC1538747

[77] Amarasekara NR, Swamy AS, Paudel SK, Jiang W, Li K, Shen C, et al. Hypervirulent clonal complex (CC) of *Listeria monocytogenes* in fresh produce from urban communities. Front Microbiol. 2024;15:1307610. 10.3389/fmicb.2024.1307610. 38348192 10.3389/fmicb.2024.1307610 PMC10859469

[78] Brown P, Hernandez K, Parsons C, Chen Y, Gould N, DePerno CS, et al. <article-title update="added"> Tetracycline resistance in *Listeria monocytogenes* and *L. innocua* from wild black bears ( *Ursus americanus* ) in the United States is mediated by novel transposable elements. Appl Environ Microbiol. 2023;89:e01205–23. 10.1128/aem.01205-23. 37888979 10.1128/aem.01205-23 PMC10686073

[79] Luque-Sastre L, Arroyo C, Fox EM, McMahon BJ, Bai L, Li F, et al. Antimicrobial resistance in *Listeria* species. Microbiol Spectr. 2018;6(4):6.4.19. 10.1128/microbiolspec.ARBA-0031-2017. 10.1128/microbiolspec.arba-0031-2017 PMC11633604 30027884

[80] Noll M, Kleta S, Al Dahouk S. Antibiotic susceptibility of 259 *Listeria monocytogenes* strains isolated from food, food-processing plants and human samples in Germany. J Infect Public Health. 2018;11:572–7. 10.1016/j.jiph.2017.12.007. 29287806 10.1016/j.jiph.2017.12.007

[81] Moura A, Leclercq A, Vales G, Tessaud-Rita N, Bracq-Dieye H, Thouvenot P, et al. Phenotypic and genotypic antimicrobial resistance of *Listeria monocytogenes*: an observational study in France. The Lancet Regional Health - Europe. 2024;37:100800. 10.1016/j.lanepe.2023.100800. 38362545 10.1016/j.lanepe.2023.100800 PMC10866989

[82] Hof H, Nichterlein T, Kretschmar M. Management of listeriosis. Clin Microbiol Rev. 1997;10:345–57. 10.1128/CMR.10.2.345. 10.1128/cmr.10.2.345 PMC172923 9105758

